# The value of the UK Clinical Aptitude Test in predicting pre-clinical performance: a prospective cohort study at Nottingham Medical School

**DOI:** 10.1186/1472-6920-10-55

**Published:** 2010-07-28

**Authors:** Janet Yates, David James

**Affiliations:** 1 Medical Education Unit, University of Nottingham Medical School, Queen's Medical Centre, Nottingham NG7 2UH, UK

## Abstract

**Background:**

The UK Clinical Aptitude Test (UKCAT) was introduced in 2006 as an additional tool for the selection of medical students. It tests mental ability in four distinct domains (Quantitative Reasoning, Verbal Reasoning, Abstract Reasoning, and Decision Analysis), and the results are available to students and admissions panels in advance of the selection process. As yet the predictive validity of the test against course performance is largely unknown.

The study objective was to determine whether UKCAT scores predict performance during the first two years of the 5-year undergraduate medical course at Nottingham.

**Methods:**

We studied a single cohort of students, who entered Nottingham Medical School in October 2007 and had taken the UKCAT. We used linear regression analysis to identify independent predictors of marks for different parts of the 2-year preclinical course.

**Results:**

Data were available for 204/260 (78%) of the entry cohort. The UKCAT total score had little predictive value. Quantitative Reasoning was a significant independent predictor of course marks in Theme A ('The Cell'), (p = 0.005), and Verbal Reasoning predicted Theme C ('The Community') (p < 0.001), but otherwise the effects were slight or non-existent.

**Conclusion:**

This limited study from a single entry cohort at one medical school suggests that the predictive value of the UKCAT, particularly the total score, is low. Section scores may predict success in specific types of course assessment.

The ultimate test of validity will not be available for some years, when current cohorts of students graduate. However, if this test of mental ability does not predict preclinical performance, it is arguably less likely to predict the outcome in the clinical years. Further research from medical schools with different types of curriculum and assessment is needed, with longitudinal studies throughout the course.

## Background

### The need for a new admissions test for medicine in the UK

There has been on ongoing debate for many years concerning the best ways of selecting medical students. Traditionally, UK medical schools relied primarily on academic achievement because the course is academically demanding. Prior academic achievement has been shown to predict success, both on the course and beyond [1-4]. However, there is an increasing demand for fairer and more transparent criteria to be developed[5]. There have been three main drivers for this: a need to recruit individuals with the personal attributes desirable in a health professional [6]; moves to 'widen participation' in medicine by attracting students from deprived or minority backgrounds who may currently fail to apply or be accepted [7]; and the fact that increasing numbers of children obtain top grades in school examinations, which means that is difficult to discriminate adequately between them on academic grounds[8]. Various medical schools have tried to develop new structures for their admissions processes in order to meet these aims [9-11], but huge individual variations remain[12].

In response to these demands, a consortium of 23 medical and dental schools in the UK introduced the UK Clinical Aptitude Test (UKCAT) in 2006[13]. This is an online test which aims to satisfy the demand for a fairer system. The UKCAT examines cognitive ability, but not acquired knowledge. Ultimately it will include some aspects of personality testing, but this section is still under development[14].

### The use of the UKCAT at Nottingham

The UKCAT was introduced in advance of any research into its potential relationship with academic performance. The development of the test by the UKCAT Board included detailed analysis of all candidates' performance, to ensure that there was minimal inherent socio-economic bias[15]. However, the Board did not make any recommendations as to how the test should be used in the admissions process; this decision was left to individual medical schools. (personal communication with UKCAT Board)

In Nottingham, the selection process for applicants in 2006-07 utilised the scores from the UKCAT sub-tests as shown in Table 1. The total score from this procedure was used to aid the selection of candidates, taking into account their domicile (for quota reasons) and allowing for non-standard examinations or UKCAT exemptions. Candidates were then invited to attend a semi-structured interview, assessing motivation, insight, empathy, and communication skills, which finally determined whether they were offered a place.

**Table 1:** Scoring system for medical school applicants

Criterion	Scoring system	Maximum score
Total score for A* and A grade passes at GCSE †	A* 2, A = 1	no maximum but unlikely to exceed 24, equivalent to 12 A* passes
Online questionnaire and tick-boxes, assessing extra-curricular activities and aptitudes ‡	Marked electronically to agreed standards	29
Personal Statement, assessed for overall impression of motivation, insight through work experience, and being a well-rounded individual	Excellent = 12, Good = 8, Below average = 4	12
UKCAT results in four cognitive domains giving four marks of 300-900 points	Scaled to 9 points per domain	36

† GCSE = General Certificate of Secondary Education, taken at age 15-16

‡ questions derived from the GMC's 'Duties of a Doctor' [23]

### Current evidence on the predictive validity of the UKCAT

The first four sub-tests of the UKCAT were designed to test cognitive ability. It therefore has the potential to differ from traditional academic tests of knowledge (as in school-leaving examinations such as A levels) which are known to exhibit socio-economic bias. As yet, there is little published evidence on its relationship with A levels nor on its predictive value for course progress. The first large study to examine the UKCAT in relation to A levels has shown that, for a sub-group of applicants who passed in three or more subjects, there was a an overall correlation between the scores achieved for both[16]. This applied to sub-test and total UKCAT scores. Socio-economic bias was slightly reduced but still present. This suggests that the UKCAT provides a reasonable proxy for A levels in this group of applicants, but not a major advantage in terms of selecting applicants who are not reaching their potential at school.

One Consortium medical school (Aberdeen) which did not use the UKCAT in its selection process has examined the correlations between UKCAT scores and its own selection process[17]. This included short-listing on the basis of academic achievements and the UCAS form, followed by an interview. The authors found only weak correlations and suggested that the UKCAT may be measuring different traits or aptitudes to the conventional selection processes in their medical school.

That school, together with another (Dundee) in which UKCAT scores were used only to determine offers in borderline decisions, has also examined UKCAT scores in relation to the Year-1 progress of students[18], and found no significant relationships.

Against this background we have investigated the relationships between UKCAT scores and the progress of a single entry cohort of students, in the first two years of the 5-year undergraduate course at Nottingham

## Methods

### Data preparation

#### Medical school entrants 2007

We used routinely-collected information to provide basic socio-demographic information for the students, including:

• Student ID number (used for subsequent linkage to course progress data)

• Sex

• DoB (used to calculate age on 01/10/2007)

• Domicile, as Home or European Union/Overseas.

• Self-declared ethnicity (recoded to White/non-White/not known)

The datafile included scores from the UKCAT. The marks for the four sections of the test were scored out of 900 and adjusted by Pearson VUE to provide a population mean of 600[15]. The total score was the sum of the adjusted sections, with a population mean of 2400 and a maximum of 3600.

We also obtained information on recent school examination results (A levels), which are provided routinely to the University by the University and College Admission Service (UCAS). A-level pass grades are awarded tariff points on the scale of A = 120, B = 100 etc, and we used these to generate information on the number of subjects passed and the average tariff score. We included the category of last recorded schooling, as provided routinely to the University by UCAS. This was recoded into three groups, Selective (Independent, Grammar or Grant Maintained), non-Selective (Comprehensive or Sixth Form College), or Unknown (ex-University or undocumented schooling).

#### The study group

Nottingham medical students who had taken the UKCAT and given written consent for their data to be used in anonymised research were designated as the study group. The Consent Form used is shown in Additional File 1.

#### Year 1 and Year 2 course progress data

The course at Nottingham is modular, with each module being awarded credits. During the first two (largely pre-clinical) years the topics are divided amongst four Themes: 'A' (the Cell), 'B' (the Person), 'C' (the Community), and 'D' (personal and professional development). Theme A, Molecular Medicine and Clinical Laboratory Sciences, is assessed predominantly by multiple choice questions in various formats. Theme B covers the structure and function of the human body, and is assessed with a mixture of online, written and practical examinations. Theme C, comprising Behavioural Sciences, Public Health, and Epidemiology, is assessed with a combination of written, online, oral presentation and coursework. Theme D includes various assessments of practical and communication skills including an OSCE (Observed Structured Clinical Examination) at the end of each year. Full details of the Schedule of Assessments are given in Additional File 2.

Average marks for each Theme were calculated for each year separately and for both years combined. The Theme D OSCE mark is included in the Theme average but also shown separately.

#### Ethical approval

Formal ethical approval was not required for this analysis of anonymised, aggregated, routinely-collected data, which is regarded as audit.

### Data Analysis

We used SPSS v17 for data analysis. The marks for course progress and scores for the UKCAT were checked for normality of distribution. Apart from that for UKCAT Quantitative Reasoning, which had a 'spike' and was slightly non-normal as indicated by the K-S statistic (p 0.002), all were normal, therefore parametric statistics were used (t-tests and Pearson Correlation coefficients for univariate comparisons, and linear regression for multivariate analysis).

The analysis consisted of:

1. basic descriptive analysis of whole cohort who commenced the course in 2007, and of the study and non-study groups, including univariate comparison of socio-demographics.

and for the study group:

2. correlation matrix for UKCAT scores & course progress data

3. univariate analysis of course progress against socio-demographic variables & UKCAT scores

4. Hierarchical multivariate linear regression of socio-demographic variables and UKCAT scores, entered in two blocks, against course progress to identify independent predictors. The regression was carried out using UKCAT sectional scores and repeated with the total score.

## Results

### The 2007 entry cohort and the study group

260 students commenced the 5-year undergraduate course in October 2007. The study group comprised 204 (78%) who had taken the UKCAT and given consent for their data to be used. In the non-study group, 10 had taken the UKCAT but not given consent, and the remaining 46 had not taken the test. (30 of these students were deferred entries from 2006. Of the remaining 16, seven were enrolled automatically after completing a Foundation programme, two were re-starting the course, six were Thai students completing a parallel course, and one had been exempted for reasons not known to us). Table 2 summarises the socio-demographic characteristics of the study and non-study groups. There were no significant differences between the two groups (Chi-square tests). Since almost all students were aged under 21, the variable for 'maturity' was not used in subsequent analyses.

**Table 2:** Socio-demographic characteristics of the 2007 entry cohort

	Entire entry cohort		study group		non-study group	
	n = 260	%	n = 204	%	n = 56	%
Female	158	61	124	61	34	61
Male	102		80		22	
						
Not mature (< 21)	257	99	203	100	54	96
Mature (> = 21)	3		1		2	
						
Home	222	85	176	86	46	82
EU or O/S	38		28		10	
						
White	160	67*	129	66*	31	70*
Non-White	79		66		13	
Unknown	21		9		12	
						
Selective schooling	144	66*	124	66*	20	67*
Non-selective schooling	75		65		10	
Unknown	41		15		26	
						

* % shown is calculated for those with known information

The UKCAT scores (mean and SD) for the study group were:

Verbal Reasoning   629 ± 72

Quantitative Reasoning   637 ± 61

Abstract Reasoning   637 ± 74

Decision Analysis   643 ± 94

Total score   2543 ± 198

Recent school examination (A-level) results were known for 193 (95%) of the study group. The remaining 11 had taken the International Baccalaureate (6), had a previous degree (2), or had other qualifications (3). Of the 193, 154 (80%) had obtained an A grade for all their subjects and therefore had an average tariff score of 120. Of the remaining 39, only 2 had an average tariff of less than 110. We therefore did not use the A-level tariff as a predictor variable in this study, since it would have had little discriminatory ability.

Full examination marks for the first two years of study were available for 195/204 students. Of the remaining nine, four had transferred out of the medical course voluntarily to study other subjects, three had transferred within the course to the BSc degree, and two had not taken all their examinations for other reasons, such as illness.

### Correlation between UKCAT scores and course progress

We first examined the correlation between Theme marks in Year 1 and Year 2. The correlation matrix is shown in Table 3 and shows a highly significant relationship (r = 0.3 - 0.8, p < 0.001) between marks for each Theme across the two years. We therefore used the overall Theme average for the remaining analysis.

**Table 3:** Correlation matrix between Theme averages in Year 1 and Year 2

		Theme A average for Yr 1	Theme B average for Yr 1	Theme C average for Yr 1	Theme D average for Yr 1	Theme D OSCE average for Yr 1
	N	255	255	255	255	255
Theme A average for Yr 2	Pearson Correlation	**.615**				
	Sig. (2-tailed)	**< 0.001**				
	N	199				
Theme B average for Yr 2	Pearson Correlation	.734	**.788**			
	Sig. (2-tailed)	< 0.001	**< 0.001**			
	N	198	198			
Theme C average for Yr 2	Pearson Correlation	.223	.241	**.426**		
	Sig. (2-tailed)	0.002	0.001	**< 0.001**		
	N	199	199	199		
Theme D average for Yr 2	Pearson Correlation	.262	.374	.403	**.355**	
	Sig. (2-tailed)	< 0.001	< 0.001	< 0.001	**< 0.001**	
	N	196	196	196	196	
Theme D OSCE for Yr 2	Pearson Correlation	.194	.271	.349	.310	**.323**
	Sig. (2-tailed)	0.007	< 0.001	< 0.001	< 0.001	**< 0.001**
	N	195	195	195	195	195

Table 4 shows the correlation matrix between the overall Theme averages and the UKCAT scores. There were statistically significant relationships between sub-tests of the UKCAT, particularly Verbal with Quantitative Reasoning (p = 0.002), and Abstract Reasoning and Decision Analysis (p < 0.001). However, the correlation coefficient was less than 0.3 in all cases.

**Table 4:** Correlation matrix between UKCAT scores and Theme averages for the first two years

		UKCAT VR	UKCAT QR	UKCAT AR	UKCAT DA	UKCAT Total	Theme A average	Theme B average	Theme C average	Theme D average	Theme D OSCE average
UKCAT VR	Pearson Correlation	1									
	Sig. (2-tailed)										
	N	204									
UKCAT QR	Pearson Correlation	.221**	1								
	Sig. (2-tailed)	**0.002**									
	N	204	204								
UKCAT AR	Pearson Correlation	0.116	.199**	1							
	Sig. (2-tailed)	0.100	**0.004**								
	N	203	203	203 †							
UKCAT DA	Pearson Correlation	.157*	.190**	.264**	1						
	Sig. (2-tailed)	0.025	**0.007**	**<0.001**							
	N	204	204	203	204						
UKCAT Total	Pearson Correlation	.557**	.546**	.625**	.720**	1					
	Sig. (2-tailed)	**<0.001**	**<0.001**	**<0.001**	**<0.001**						
	N	204	204	203	204	204					
Theme A average	Pearson Correlation	.189**	.240**	0.038	0.06	.211**	1				
	Sig. (2-tailed)	**0.008**	**0.001**	0.597	0.396	**0.003**					
	N	199	199	198	199	199	199				
Theme B average	Pearson Correlation	0.133	.152*	0.003	0.036	0.126	.886**	1			
	Sig. (2-tailed)	0.063	0.032	0.961	0.619	0.078	**<0.001**				
	N	198	198	197	198	198	198	198			
Theme C average	Pearson Correlation	.319**	0.073	0.117	0.066	.232**	.566**	.528**	1		
	Sig. (2-tailed)	**<0.001**	0.308	0.102	0.352	**0.001**	**<0.001**	**<0.001**			
	N	199	199	198	199	199	199	198	199		
Theme D average	Pearson Correlation	0.003	0.018	-0.028	-.155*	-0.085	.367**	.463**	.463**	1	
	Sig. (2-tailed)	0.972	0.798	0.694	0.030	0.237	**<0.001**	**<0.001**	**<0.001**		
	N	196	196	195	196	196	196	196	196	196	
Theme D OSCE average	Pearson Correlation	0.039	0.066	0	-0.085	-0.014	.274**	.336**	.375**	.704**	1
	Sig. (2-tailed)	0.584	0.361	0.991	0.239	0.849	**<0.001**	**<0.001**	**<0.001**	**<0.001**	
	N	195	195	194	195	195	195	195	195	195	195

VR = Verbal Reasoning; QR = Quantitative Reasoning; AR = Abstract Reasoning; DA = Decision Analysis. †1 student had no score provided for Abstract Reasoning, reason unknown

Within the Themes alone there were stronger correlations (p < 0.001 in all cases). The coefficients were large between the knowledge-based assessments (A and B, r = 0.87) and weakest between Theme A and the OSCE (r = 0.27).

There were only three modest correlations between the UKCAT sub-tests and the Theme marks: Verbal Reasoning and Theme A and Theme C, and Quantitative Reasoning with Theme A. These were relatively weak (r = 0.32 or less). There were no significant correlations between UKCAT total score and the Themes.

### Univariate analysis of socio-demographic variables against UKCAT scores and course progress

We used t-tests to examine the effects of socio-demographic variables (sex, ethnicity, domicile and schooling) on UKCAT scores and Theme averages. The Bonferroni correction for multiple comparisons would suggest that significance values larger than p = 0.01 are not of practical importance.

Table 5 summarises the few statistically significant differences that were found. The total UKCAT score was little affected by socio-demographic variables, with a weak positive influence of Home domicile and White ethnicity. There were scattered effects on sub-scores. On the course, Theme C was the most affected, with poorer performance by males but a positive influence of White ethnicity and Home domicile.

**Table 5:** Significant univariate effects (t-tests) of socio-demographic variables on UKCAT scores and Theme averages (Year 1 plus Year 2)

Socio-demographic variable	Test parameter	Mean	SD	t	p
**Sex**					
Male	UKCAT QR	654	51.5	3.24	0.001
Female		626	64.6		
					
Male	Theme C	62	7.4	-3.42	0.001
Female		65	7.3		
					
Male	Theme D	65	6.7	-3.40	0.001
Female		68	6.0		
					
**Domicile**					
Home (UK)	UKCAT VR	637	70.5	4.12	< 0.001
EU or Overseas		579	65.0		
					
Home (UK)	UKCAT AR	644	71.6	3.38	0.001
EU or Overseas		594	72.2		
					
Home (UK)	UKCAT Total score	2556	190.1	2.38	0.02
EU or Overseas		2461	231.8		
					
Home (UK)	Theme C	65	7.3	2.91	0.004
EU or Overseas		60	7.7		
					
**Ethnicity**					
White	UKCAT VR	644	59.8	3.73 *	< 0.001
Non-White		600	86.5		
					
White	UKCAT Total score	2571	171.5	2.10 *	0.04
Non-White		2502	235.1		
					
White	Theme C	66	7.1	3.85 *	0.001
Non-White		61	7.5		
					
**Schooling**					
Selective	Theme C	63	7.3	- 2.52	0.01
Non-Selective		66	7.5		
					
Selective	Theme D OSCE	63	9.8	- 2.2	0.03
Non-Selective		66	9.6		

Each socio-demographic variable was tested against all UKCAT scores and Theme averages but only the statistically significant values are shown.

* Levene's test, unequal variances.

### Multivariate analysis

Table 6 summarises the statistically significant results from the hierarchical multivariate linear regressions. All results significant at p < 0.05 are shown, although those with p > 0.01 are unlikely to be of practical importance as described above. As expected from the univariate analyses, there were few independent predictors of Theme scores.

**Table 6:** Significant independent predictors of course performance (hierarchical multivariate linear regression analysis)

Outcome variable	Predictor variable block ^†^	R ^2^	Δ R^2 ‡^	Significant predictors	Beta	t	P ^§^
	**Including UKCAT total score**						
Theme A	1 (socio-demographic)	0.02	0.04	None			
	2 (UKCAT total score)	0.04	0.03 *	UKCAT total score	0.17	2.24	0.03
							
Theme B	1 (socio-demographic)	0.01	0.03	None			
	2 (UKCAT total score)	0.02	0.02	None			
							
Theme C	1 (socio-demographic)	0.12	0.14 ***	Male sex	-0.23	-3.22	0.002
				White Ethnicity	0.24	3.07	0.002
	2 (UKCAT total score)	0.15	0.03 *	Male sex	-0.25	-3.55	< 0.001
				White Ethnicity	0.22	2.83	0.005
				UKCAT total score	0.18	2.54	0.012
							
Theme D	1 (socio-demographic)	0.03	0.06 *	Male sex	-0.17	-2.31	0.02
	2 (UKCAT total score)	0.04	0.01	Male sex	-0.16	-2.12	0.04
							
Theme D OSCE	1 (socio-demographic)	0.01	0.03	None			
	2 (UKCAT total score)	0.00	0.00	None			
							
	**Including UKCAT sub-test scores**						
Theme A	1 (socio-demographic)	0.02	0.04	None			
	2 (UKCAT sub-test scores)	0.09	0.09 **	UK student	-0.18	-2.22	0.028
				Selective Schooling	-0.18	-2.39	0.018
				UKCAT Verbal Reasoning	0.19	2.41	0.017
				UKCAT Quantitative Reasoning	0.22	2.85	0.005
							
Theme B	1 (socio-demographic)	0.01	0.03	None			
	2 (UKCAT sub-test scores)	0.05	0.06 *	Selective Schooling	-0.16	-2.13	0.034
				UKCAT Verbal Reasoning	0.20	2.54	0.012
							
Theme C	1 (socio-demographic)	0.12	0.14 ***	Male sex	-0.23	-3.22	0.002
				White Ethnicity	0.24	3.07	0.002
	2 (UKCAT sub-test scores)	0.19	0.08 **	Male sex	-0.26	-3.63	< 0.001
				White Ethnicity	0.18	2.38	0.019
				Selective Schooling	-.016	-2.24	0.026
				UKCAT Verbal Reasoning	0.28	3.86	< 0.001
							
Theme D	1 (socio-demographic)	0.03	0.06 *	Male sex	-0.17	-2.31	0.02
	2 (UKCAT sub-test scores)	0.06	0.04	Male sex	-0.18	-2.37	0.019
				Selective Schooling	-0.16	-2.07	0.04
				UKCAT Decision Analysis	-.21	-2.67	0.008
							
Theme D OSCE	1 (socio-demographic)	0.03	0.03	None			
	2 (UKCAT sub-test scores)	0.05	0.03	None			

^† ^denotes the successive addition of variable blocks to the hierarchical regression, as defined in the Methods

^‡ ^a significant value in this column it indicates that the additional block of variables adds significantly (in terms of variance) to the prediction of the outcome variable. * p < .05, ** p < .01, *** p < .001

§ using the Bonferroni correction for multiple comparisons, p = < 0.01 for significance

VR = Verbal Reasoning, QR = Quantitative Reasoning, AR = Abstract Reasoning, DA = Decision Analysis

In the upper part of Table 6, UKCAT total scores are used in Block 2. It is evident that neither socio-demographic variables nor UKCAT have substantial predictive value for the Theme averages, with the exception of Theme C, in which male sex has a strong negative influence and White ethnicity a positive one. Further examination of the data showed that these differences lay primarily in the Behavioural Sciences module (year 1) and Epidemiology in Practice (year 2) respectively (p < 0.001 in both cases, data not shown). The UKCAT total score has an additional weak positive relationship with Themes A and C and adds a small amount of variance to the model.

In the lower part of Table 6, UKCAT sub-test scores are used in Block 2. Of note are the influences of Quantitative Reasoning in Theme A and Verbal Reasoning in Theme C, with an additional weak effect of Verbal Reasoning in Theme A. Male sex is a strong negative predictor in Theme C, but otherwise the effects of socio-demographic variables are modest.

## Discussion

This small study suggests the UKCAT has very limited predictive value for the performance over the first two years of preclinical study at Nottingham. The total score had only very modest correlation with Themes A and C. This effect appeared to be exerted via Quantitative Reasoning in Theme A, and Verbal Reasoning in Theme C. Socio-demographic variables also had little influence, apart from male sex and white ethnicity in Theme C.

### Strengths and weaknesses of the study

This paper adds to the currently sparse evidence relating to the UKCAT. Although the study group includes only 78% of the intake cohort, these students did not differ in socio-demographic terms from their peers, who had either not taken the UKCAT or not given permission for their data to be used. Thus there is no *a priori *reason to suppose that our findings are unrepresentative.

We had to exclude nine students (4% of the study group) from the statistical analyses because they did not have full datasets. The reasons were varied, and included academic difficulties, health problems, and personal issues. With such small numbers we decided that it was not appropriate to examine whether overwhelming academic failure was related to UKCAT score, although this is a potentially important issue.

We did not attempt to compare students' performance in the UKCAT with their school leaving examinations. As noted, only one publication to date has looked at this issue[16]. That study investigated the sub-group of medical school applicants who went on to achieve at least three passes at A level, and demonstrated a modest correlation between UKCAT scores and A levels. In the current study, so many students had average A-level tariff scores at the maximum (120) that we could not use them in a comparable analysis.

The variance contributed by the explanatory variables was small. This is in line with other research at Nottingham[19].

### Socio-demographic predictors of pre-clinical performance

Socio-demographic influences on performance were generally slight, and corresponded with previous research both at Nottingham and elsewhere[19-21]. We have no ready explanation for the poorer performance of males in Behavioural Sciences, and of non-White students in Epidemiology. These findings merit further internal investigation.

### Differential effects of the UKCAT section scores

Our study suggests that the total UKCAT score has little predictive relationship with preclinical performance. It must be remembered that students on the course are already a highly selected group, and their UKCAT scores probably lie within a relatively small range, compared to the wider pool of applicants. Although school examination results have been shown to relate to academic performance on the course in the past [1,2,4], we might speculate that it is less likely that this correlation would be shown now, when an increasing majority of medical students have top grades[8]. Perhaps we should not expect UKCAT scores to correlate highly either, in the select group of students who have achieved admission. However, an overall relationship between UKCAT scores and course progress might still be observed if the UKCAT is able to identify students with good potential abilities. The section scores may prove more sensitive in this respect.

The modest relationship of UKCAT Quantitative Reasoning with Theme A is not unexpected, since that part of the course is the most 'scientific' in terms of course content and assessment. The ability of Verbal Reasoning to predict performance in Theme C is interesting. The types of assessment are mixed and include oral presentation and an essay, as well as short-answer and multiple choice questions. The subject matter perhaps requires a deeper level of thought and understanding, and better articulation, than Themes A and B, which are based more on acquired knowledge. Theme D is assesses through coursework, practical communication and clinical skills, so it is unsurprising that clear cognitive predictors did not emerge, as other factors such as personality are likely to be more important. It is of note that the correlation coefficients between Themes A and B in years one and two are stronger than those between Themes C and D and the OSCEs. We speculate that this could reflect that the format and content of the respective assessments (see Supplement 1). Themes A and B comprise science knowledge in both years and use similar assessments, whereas the content and assessments of Themes C and D are more varied. Certainly the standard of clinical performance required for year two OSCEs is intended to be higher than year 1.

## Conclusions

This limited study suggests that the predictive value of the UKCAT, particularly the total score, is low in the pre-clinical course at Nottingham. The ultimate test of validity will not be available for some years, when current cohorts of students graduate. However, if this test of mental ability does not predict preclinical performance, it is perhaps even less likely to predict the outcome in the clinical years. Research elsewhere has suggested a multi-faceted approach to selection would be best, including personality testing[22]., since a 'good doctor' requires many skills and abilities besides academic prowess. The planned non-cognitive part of the UKCAT (Section 5, currently undergoing trials [14]) may add value in this respect. Further longitudinal studies are required, involving consecutive year-groups from other medical schools with different types of curriculum and assessment.

## Competing interests

DJ and JY conducted research on the UKCAT national database during 2007-08 and JY was employed part-time by UKCAT for this purpose. This analysis is entirely separate, using data supplied by UKCAT to Nottingham for institutional purposes. Neither author currently works with or for UKCAT in any capacity, nor expects to do so. We therefore declare that we have no competing interests.

## Authors' contributions

Both authors planned the study, contributed to the interpretation of the data and the writing of the paper, and approved the final draft. JY prepared and analysed the data and wrote the first draft.

## Authors' information

DJ, Emeritus Professor of Feto-maternal Medicine, was Foundation Director of Medical Education at Nottingham from 2002-2008. He has published several papers on the processes for selection of medical students and their subsequent progress on the course He has played a significant role in shaping admissions policy at Nottingham.

JY has been Research Fellow in Medical Education at Nottingham since 2003, focussing on student progress, particularly those students who underperform or fail on the course.

## Funding

JY is funded by the Service Increment for Teaching (SIFT)

## Pre-publication history

The pre-publication history for this paper can be accessed here:

http://www.biomedcentral.com/1472-6920/10/55/prepub

## Supplementary Material

Additional file 1 **Consent Form issued by students in October 2007**. Click here for file

Additional file 2 **Schedules of Assessment**. Click here for file
