# Supplementary material for: The value of the UK Clinical Aptitude Test in predicting pre-clinical performance: a prospective cohort study at Nottingham Medical School
Source: BMC Med Educ. 2010 Jul 28;10:55. doi: 10.1186/1472-6920-10-55 (PMC2922293; doi:10.1186/1472-6920-10-55)
Supplement: Additional file 1 — Consent Form issued by students in October 2007. [file 1472-6920-10-55-S1.DOC]

**Additional file 1: Consent Form issued to students in October 2007**

Students entering the course in 2007 were asked if they would provide signed consent for their UKCAT data to be used in future research studies.

**Consent Form for UKCAT**

You sat the UKCAT prior to your admission to Nottingham Medical School. It is very important that we are able to follow up those who have taken the UKCAT to obtain more information regarding the value of the different components of the test by comparing your scores with your progress through Medical School. This will have benefits in the future in that not only could these tests be used in selection but they could aid counselling when students have difficulties in Medical School. They might also be used to identify those who will do well in particular areas of Medicine.

You will not be identified by name during these studies. You will be allocated a matriculation number on admission to the Medical School. We can then relate this to the UCAS number that you held at the time of sitting the test. At no point will you be identified by name as any of your data will always be analysed with those of others and will not be looked at individually. All data are held on confidential, anonymised, data-bases in secure conditions. This also means that the data from the UKCAT cannot be used as part of your assessment once you have entered Medical school.

If you agree to help, please sign the form below.

Signed:…………………………………………………………………..

Print Name:……………………………………………………………

Date:…………………………………………………………………….
