# Supplementary material for: The value of the UK Clinical Aptitude Test in predicting pre-clinical performance: a prospective cohort study at Nottingham Medical School
Source: BMC Med Educ. 2010 Jul 28;10:55. doi: 10.1186/1472-6920-10-55 (PMC2922293; doi:10.1186/1472-6920-10-55)
Supplement: Additional file 2 — Schedules of Assessment. [file 1472-6920-10-55-S2.DOC]

**Additional file 2: Schedules of Assessment**

This document summarises the examinations taken by students in 2007-08 (year 1) and 2008-09 (year 2). There are additional optional modules taken in Semester 4 but these are not shown since the marks do not contribute to the Theme marks.

**Year 1**

| **YEAR 1 CORE & OPTIONS 2006-07 AUTUMN (Semester 1)** | | | | |
| --- | --- | --- | --- | --- |
| **CORE Modules** | **CODE** | **CREDITS** | **THEME** | **CONTENT OF ASSESSMENT** |
| Molecular Basis of Medicine | A11MBM | 15 | A | 100 MCQs to be answered in 2 hours |
| Structure, function and pharmacology of excitable tissues | A11EXT | 10 | B | 1.5 hour examination with 100 MCQs questions (includes factual recall, problem solving, and logical reasoning tasks). |
| Human Development & Tissue Differentiation | A11HDT | 10 | B | **Written exam** 1 50% 1 hour exam of 3 short essays  **Online exam 2** 50% 1 hour practical exam |
| Behavioural Sciences | A11BHS | 10 | C | a)Written exam (70%) 1.0 hour unseen paper consisting of 4 questions based on the taught course and requiring short notes answers.  b)Oral presentation plus handout (25%).  C) Presentation – peer contribution. (Tutor moderated) (5%) |
| Public Health Medicine 1 | A11PH1 | 5 | C | Written exam of 6 short answers 45 Mins |
| **YEAR 1 CORE & OPTIONS 2006-07 SPRING (Semester 2)** | | | | |
| Clinical Laboratory Sciences (I) | A11CLS | 10 | A | 100 MCQ 2 Hours |
| Human Development Structure and Function (I) | A11SF1 | 20 | B | **Coursework 1** 10% Dissection assessment  **Inclass exam 1** 50% 45 min practical exam.  **Inclass exam 2** 20% 45 min T/F/A exam  **Exam 3** 20% 1 hour exam of 2 short essays |
| Cardiovascular, Respiratory & Haematology | A11CRH | 20 | B | 2.5 hours 100 T/F/A (80%) and 2 short answers (2 x 10%), inlcudes Anatomy, Histology and Physiology |
| **YEAR 1 CORE & OPTIONS 2006-07 Year-long modules** | | | | |
| Early Clinical & Professional Development (I) | A11PD1 | 15 | D | 1. 50% 45min OSCE exam testing at least six clinical skills related to the module 2. b) 50% written coursework consisting of satisfactory completion of self-directed learning tasks based on clinical visits. **Must be passed.** |
| Communication Skills (I) | A11CS1 | 5 | D | Transcription and evaluation of taped patient interview. **Must be passed.** |

| **Key** |  |  |
| --- | --- | --- |
| MCQ | = | Multiple Choice Question |
| T/F/A | = | True/False/Abstain machine markable tests |
| SW | = | Single word answers |
| SP | = | Single phrase answers |
| SA | = | Short answers |
| SE | = | Short essays |
| OSCE | = | Objective structured clinical examination |

**Year 2**

| **Year 2 CORE & OPTIONS 2007-08 Autumn (Semester 3)** | | | | |
| --- | --- | --- | --- | --- |
| **CORE Modules** | **CODE** | **CREDITS** | **THEME** | **CONTENT OF ASSESSMENT** |
| Human Development Structure and Function (II) | A12SF2 | 20 | B | 1. **Coursework 1** 10% Dissection assessment 2. **Exam 3** 20% 1 hour exam of 2 short essays 3. **Inclass exam 1** 50% 45 min practical exam 4. **Inclass exam 2** 20% 50 T/F/A question exam (45min) |
| Alimentary System & Nutrition | A12ASN | 10 | B | 1. Exam 1 80% 100 T/F/A question exam. 1 Hour 30 Mins 2. Exam 2 20% Anatomy/histology spotter (15mins) |
| Renal and Endocrine Systems | A12REN | 10 | B | 1 x 2.0h 130 T/F/A exam |
| **Year 2 CORE & OPTIONS 2007-08 Spring (Semester 4)** | | | | |
| Functional and Behavioural Neuroscience | A12FBN | 15 | B | 2hr exam:   1. 75 T/F/A question exam (50%) 2. one seen case history with 6 questions (50%) |
| General & Biochemical Pharmacology | A12GBP | 5 | B | 1hr 50 T/F/A question exam |
| **Year 2 CORE & OPTIONS 2007-08 Year-long modules** | | | | |
| Clinical Laboratory Sciences (II) | A12CLS | 20 | A | 3 hour exam:   1. 100 MCQ 2. 6 structured short notes |
| Public Health Medicine (II) | A12PH2 | 5 | C | Coursework 1 50% U: 1,000 word essay.  Coursework 2 50% S: 1,000 word essay |
| Early Clinical & Professional Development (II) | A12PD2 | 10 | D | 1. 50% 45min OSCE exam testing at least six clinical elements related to the module 2. 50% Written coursework consisting of satisfactory completion of self-directed learning tasks based on clinical visits **Module and OSCE must be passed.** |
| Communication Skills (II) | A12CS2 | 5 | D | 1. Coursework 1 75% Reflective portfolio. 500 word commentary on evidence related to interviews with patients. 2. Coursework 2 5% Peer assessment of DVD. Recorded interview with a simulated patient using a structured pro-forma. 3. Inclass Exam 1 20% Staff observed assessment of explanation skills.   **Module must be passed.** |

| **Key** |  |  |
| --- | --- | --- |
| **MCQ** | = | Multiple Choice Question |
| **T/F/A** | = | True/False/Abstain machine markable tests |
| **SW** | = | Single word answers |
| **SP** | = | Single phrase answers |
| **SA** | = | Short answers |
| **SE** | = | Short essays |
| **OSCE** | = | Objective structured clinical examination |
